# Growth–defense trade-offs in *Quercus variabilis* and *Quercus mongolica* seedlings under drought stress

**DOI:** 10.3389/fpls.2026.1941405

**Published:** 2026-10-07

**Authors:** Zhigang Xue, Shangyong Yin, Yuedong Guo, Kejia Cui, Lei Wu, Xingpeng He, Hao Wu, Runmei Gao, Jinhua Bai

**Affiliations:** College of Forestry, Shanxi Agricultural University, Taigu, China

**Keywords:** antioxidant defense, drought response, ecotones, non-structural carbohydrates, trait trade-offs

## Abstract

**Introduction:**

Intensified drought induced by climate change is threatening the natural regeneration of forests, yet coexisting tree species can maintain their survival and regeneration by adopting divergent carbon allocation strategies. This study compared leaf-scale drought responses of *Quercus variabilis* and *Q. mongolica* seedlings originating from the ecotone of the Zhongtiao Mountains.

**Methods:**

Multiple indicators were measured under four water regimes: control (CK), light drought (LD), moderate drought (MD), and severe drought (SD), including leaf morphology, photosynthetic and gas exchange parameters, hydraulic traits, carbon storage traits, stress defense traits, and nutrient stoichiometry. Principal component analysis (PCA) and structural equation modeling (SEM) were used to evaluate interspecific differences in drought adaptation strategies.

**Results:**

Drought suppressed leaf growth and gas exchange, lowered leaf water potential and relative water content, aggravated damage to hydraulic function, and boosted biochemical responses associated with stress defense in both species. For *Q. variabilis*, leaf non-structural carbohydrate (NSC) concentrations were tightly correlated with growth-related traits, indicating that NSCs play a vital role in sustaining growth and accumulating carbon after growth limitation, reflecting a growth-prioritized resource allocation strategy. In contrast, leaf NSCs of *Q. mongolica* seedlings exhibited stronger coordinated covariation with stomatal behavior and antioxidant defenses, suggesting that this species prioritizes defense maintenance and physiological stability under drought stress, representing a defense-prioritized resource allocation strategy.

**Discussion:**

Seedlings of *Q. variabilis* and *Q. mongolica* adopt growth-prioritized and defense-prioritized adaptation strategies under drought, respectively. Such physiological divergence serves as a physiological foundation for niche differentiation, divergent regeneration patterns, and coexistence of seedlings of the two Quercus species within the ecotone.

## 1 Introduction

Climate warming and altered precipitation patterns are increasing the frequency of droughts worldwide, thereby threatening forest ecosystems (Gebrechorkos et al., 2025). Previous studies have documented a global increase in drought-induced forest decline and tree mortality, potentially disrupting terrestrial carbon and water cycling and constraining forest regeneration (Anderegg et al., 2016; Hammond et al., 2022). Natural forest regeneration depends on sequential processes, from seed germination and seedling establishment to sapling growth and canopy recruitment (Chang-Yang et al., 2021; Harris et al., 2022). Seedlings are especially vulnerable to environmental stress and may therefore act as a key bottleneck for population regeneration and community dynamics (Lalor et al., 2023). Ecotones, as transition zones between vegetation types or climatic regions, are characterized by steep environmental gradients, fluctuating resources, and complex community composition, making them particularly sensitive to climate change and water limitation (You et al., 2021; De La Riva et al., 2023). In these systems, interspecific differences in seedling drought responses may influence regeneration success, niche differentiation, and species coexistence (MacDonald et al., 2026). As warming continues and drought intensifies, such differences may shape community regeneration patterns and help maintain species distribution boundaries (Anderegg and HilleRisLambers, 2016; Yuan et al., 2022). Understanding drought-response capacity and establishment strategies in ecotonal seedlings is therefore critical for predicting plant community regeneration, structural stability, and long-term dynamics.

Drought-induced water deficit drives coordinated shifts in plant morphology, photosynthesis, hydraulics, and stress defense, thereby mediating trade-offs between growth maintenance and defense investment (Huo et al., 2024; Wang et al., 2025). Functional traits and trait plasticity therefore provide useful indicators for interpreting drought-adaptation strategies (Kramp et al., 2022; Férriz et al., 2023). Fast-growing seedlings may benefit from rapid resource acquisition and biomass accumulation, but this strategy is often associated with weaker drought-defense capacity (Metz et al., 2023; Wang et al., 2024b). Conversely, slower-growing seedlings that invest more heavily in defense and maintenance may have greater survival potential in dry environments (Negreiros et al., 2016; Volaire et al., 2023). Under drought, stomatal closure reduces water loss but limits CO_2_ uptake and photosynthetic carbon assimilation (Zhou et al., 2014; Chen et al., 2022). Persistent water deficit may further induce hydraulic dysfunction, altered carbon metabolism, and oxidative damage, ultimately suppressing growth and, under severe stress, leading to mortality (Kono et al., 2019; Trueba et al., 2024). Nonstructural carbohydrates (NSCs) are central to plant carbon metabolism, supplying substrates for growth and respiration while supporting osmotic adjustment, antioxidant defense, and post-stress recovery (Hartmann and Trumbore, 2016; Piper et al., 2022). Thus, integrating leaf morphology, photosynthetic physiology, hydraulic status, NSC dynamics, inducible stress-defense traits, and nutrient stoichiometry may help clarify the mechanisms underlying growth–defense trade-offs during seedling adaptation to drought.

As dominant forest-forming taxa across the Northern Hemisphere, oaks (*Quercus* spp.) play key roles in regulating hydrological processes and maintaining forest ecosystem stability (Kremer and Hipp, 2020; Qazi, 2020). Temperature and precipitation are major climatic drivers of the potential distributions of *Quercus* species, and ongoing climate change is expected to shift suitable habitats for some oaks toward higher latitudes and elevations (Lin et al., 2023). *Q. variabilis* and *Q. mongolica* are widely distributed oak species in East Asian deciduous broad-leaved forests. *Q. variabilis* occurs mainly in low mountains and hilly landscapes from the warm temperate to northern subtropical zones, whereas *Q. mongolica* has a more northerly distribution center, extending from the northern temperate zone to the southern margin of the cold temperate zone (Chen et al., 2012; Zeng et al., 2015). Their ranges overlap from northern North China to southern Northeast China, forming an ecotone characterized by range-edge sensitivity and species admixture (Sun et al., 2021). Within this region, water limitation and drought stress can significantly suppress the growth and physiological performance of *Q. variabilis* and *Q. mongolica* seedlings and may consequently constrain seedling establishment and regeneration (Dong and Sang, 2012; Xu et al., 2015). Because seedling drought tolerance can strongly influence population regeneration and range-boundary persistence, comparing the drought-adaptation strategies of *Q. variabilis* and *Q. mongolica* seedlings can help clarify the mechanisms underlying oak coexistence and potential distributional shifts within this ecotone.

In recent years, studies on drought adaptation in *Q. variabilis* and *Q. mongolica* seedlings have expanded from growth responses to multiple dimensions, including physiological and biochemical responses, carbon allocation, and hydraulic regulation. However, existing studies have generally focused on selected functional traits or compared each species separately with other tree species. Relatively few studies have systematically compared the drought-adaptation strategies of the two species under a common drought gradient by integrating multidimensional functional traits related to morphology, photosynthesis and gas exchange, hydraulics, carbon storage, stress defense, and nutrient stoichiometry, while explicitly considering coordination and trade-offs among traits. Here, we exposed seedlings of both species to a drought-stress gradient and quantified functional traits spanning morphology, photosynthetic gas exchange, hydraulic function, carbon storage, stress defense, and nutrient stoichiometry. By integrating trait responses with trait–trait relationships, we sought to identify the ecophysiological mechanisms that differentiate their drought-adaptation strategies. This work may help explain niche differentiation, regeneration patterns, and oak coexistence in forest ecotones under climate change. Specifically, we asked: do *Q. variabilis* and *Q. mongolica* seedlings exhibit distinct drought-adaptation strategies within the ecotone, and which functional traits and trait associations underpin these differences?

## 2 Materials and methods

### 2.1 Study site and experimental design

The experiment was conducted at the Forestry Station of Shanxi Agricultural University in Taigu District, Jinzhong, Shanxi Province, China. Seeds of *Q. variabilis* and *Q. mongolica* were collected from natural populations in the Zhongtiao Mountains, a climatic transition region between the warm-temperate and northern subtropical zones. The region has a mean annual temperature of 10.3 °C and an annual precipitation of approximately 700 mm, with most precipitation occurring from June to September (Ning et al., 2023; **Figure 1**).

**Figure 1:**
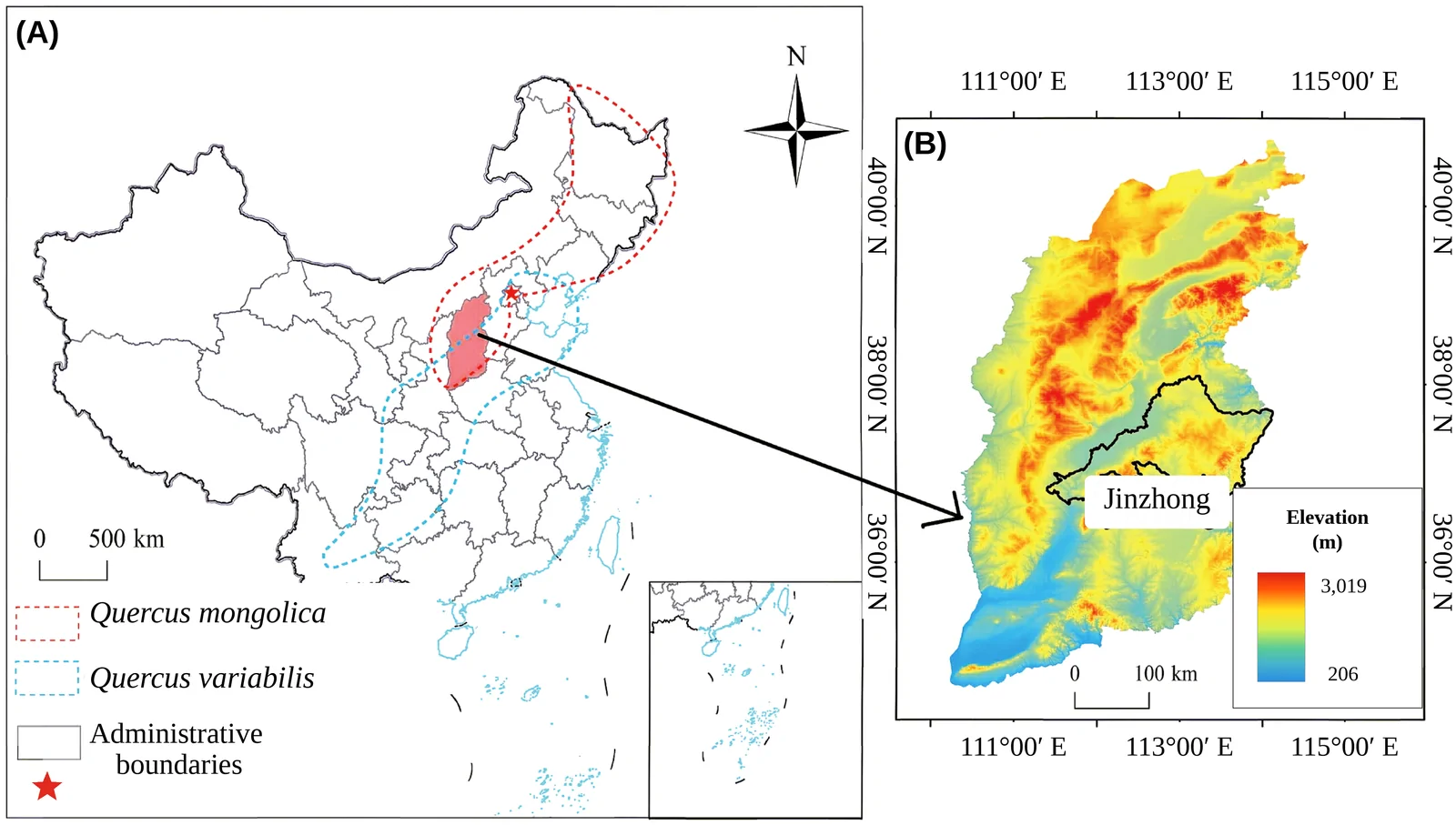
Geographic distributions of the two *Quercus* species and the location of the study site. **(A)** Geographic distribution ranges of *Q. mongolica* and Q. variabilis in China. The red and blue dashed lines indicate the distribution boundaries of *Q. mongolica* and *Q. variabilis*, respectively. **(B)** Location of the study site.

In early March 2025, seedlings of *Q. variabilis* and *Q. mongolica* were individually transplanted into plastic pots (30 cm in upper diameter, 18 cm in lower diameter, and 38 cm in height), each containing 10 kg of a substrate consisting of sandy loam and perlite mixed at a ratio of 4:1. The experiment was conducted under a transparent rain shelter located in an open, unobstructed area. Light conditions within the shelter were relatively uniform, and ventilation was adequate. After transplantation, the seedlings were watered regularly and allowed to acclimate to the pot environment for three months. Before the drought experiment, healthy seedlings that were relatively uniform in size and vigor were selected. The initial height and basal diameter of *Q. variabilis* seedlings were approximately 12–13 cm and 3.0–3.5 mm, respectively, whereas those of *Q. mongolica* seedlings were approximately 8–9 cm and 2.5–3.0 mm, respectively. Following the three-month acclimation period, drought treatments were initiated in June 2025 for seedlings of both species (Wang et al., 2023). Before the drought treatment, the field capacity (FC) of the substrate was determined using the ring-knife gravimetric method. Representative samples of the mixed substrate were fully saturated with water and allowed to drain freely under gravity for 24 h. Their wet masses were then recorded, after which the samples were oven-dried at 105 °C to constant mass and weighed again to determine their dry masses. Field capacity was calculated as the difference between the wet and dry masses divided by the dry mass and expressed as a percentage. The measured field capacity of the substrate was (26.31 ± 2.80)% (mean ± SD, n = 3).

Four soil water treatments were established based on substrate field capacity: control (CK; 80% ± 5% of FC), light drought (LD; 60% ± 5% of FC), moderate drought (MD; 40% ± 5% of FC), and severe drought (SD; 20% ± 5% of FC). For each species, 80 seedlings were randomly assigned to the four water treatments, with 20 seedlings per treatment and one seedling per pot, resulting in a total of 160 seedlings. Subsequently, pots from all combinations of species and water treatments were randomly arranged under the rain shelter. To minimize potential positional effects arising from spatial heterogeneity in light, ventilation, and microclimatic conditions, pot positions were randomly reassigned every 3 d. Drought treatments were applied from 1 June to 31 July 2025, lasting 60 d. At the beginning of the drought treatment, irrigation was withheld to allow the substrate water content to decline gradually to the target level for each treatment. Once the target water level was reached, water was added to each pot daily between 17:00 and 19:00 based on pot-mass measurements and readings from a soil moisture meter to maintain the substrate water content within the designated range for the corresponding treatment. At the end of the drought treatment, samples were transported to the laboratory for measurements of leaf morphological, photosynthetic, hydraulic, carbon-storage, stress-defense, and nutrient-stoichiometric traits.

### 2.2 Measurement of relevant traits

#### 2.2.1 Morphological traits

At the end of the drought treatment, on 31 July 2025, five seedlings of each species, *Q. variabilis* and *Q. mongolica*, were randomly selected from each drought level as biological replicates. Mature, fully expanded, intact, and pest-free functional leaves were collected from the middle-to-upper crown of each seedling and transported to the laboratory for morphological measurements. Leaf area (LA, cm^2^) was measured with an LI-3100C leaf area meter (LI-COR Biosciences, Lincoln, NE, USA). The leaves were then oven-dried to constant mass, and leaf dry weight (LDW, g) was recorded. Specific leaf area (SLA, cm^2^ g^-1^) was calculated using **Equation 1**.

(1) $$SLA=\frac{LA}{LDW}$$

where SLA is the specific leaf area (cm^2^ g^-1^), LA is the leaf area (cm^2^), and LDW is the leaf dry weight (g).

#### 2.2.2 Photosynthetic and gas-exchange parameters

Leaf gas-exchange parameters were measured with a portable photosynthesis system (LI-6400XT, LI-COR Biosciences, Lincoln, NE, USA). During measurements, the leaf chamber temperature was maintained at 25 °C. A red–blue artificial light source was used, with photosynthetically active radiation set to 1200 μmol m^-^² s^-^¹ and the gas flow rate set to 500 μmol s^-^¹. The measured parameters included net photosynthetic rate (*P_n_*), stomatal conductance (*G_s_*), and transpiration rate (*T_r_*). Instantaneous water-use efficiency (WUE) was calculated using **Equation 2**.

(2) $$WUE=\frac{P_{n}}{T_{r}}$$

where WUE is instantaneous water-use efficiency, *P_n_* is net photosynthetic rate, and *T_r_* is transpiration rate.

#### 2.2.3 Hydraulic traits

Leaf water potential was measured on 31 July 2025 using a portable plant water-potential pressure chamber. Measurements were conducted predawn, between 04:30 and 05:30, and at midday, between 12:00 and 13:00. For each treatment, five seedlings of similar size and vigor were selected, and mature functional leaves from the middle-to-upper crown of each seedling were used for measurements. Stem segments were subsequently collected from the same seedlings for measurement of stem embolism. Stem embolism was assessed using a hydraulic conductivity measurement system. Immediately after sampling, the initial hydraulic conductivity (K_h_) of each stem segment was measured. Each stem segment was then connected to a flushing device and flushed continuously with 20 mmol L^-1^ KCl solution at 0.1 MPa for 20 min to remove xylem embolism. After flushing, the stem segment was equilibrated under water for 20 min and then reconnected to the hydraulic conductivity measurement system to determine maximum hydraulic conductivity (K_h-max_, kg m s^-1^ MPa^-1^). The percentage loss of The percentage loss of hydraulic conductivity (PLC) was calculated using **Equation 3**.

(3) $$PLC=100\times \frac{K_{h-max}-K_{h}}{K_{h-max}}$$

where PLC is the percentage loss of hydraulic conductivity (%), Kh​ is the initial hydraulic conductivity before flushing, and Kh-max​ is the maximum hydraulic conductivity measured after flushing to remove embolism.

For leaf relative water content (LRWC), five mature functional leaves were collected from the middle-to-upper crown of each seedling, and their fresh weight (FW) was recorded. The leaves were then immersed in deionized water for 24 h to achieve full hydration. After surface water was gently blotted dry, turgid weight (TW) was recorded. Finally, the leaves were oven-dried at 65 °C to constant weight, and dry weight (DW) was recorded. Leaf relative water content (LRWC) was calculated using **Equation 4**.

(4) $$LRWC=\frac{FW-DW}{TW-DW}\times 100\%$$

where LRWC is leaf relative water content (%), FW is fresh weight, TW is turgid weight, and DW is dry weight.

#### 2.2.4 Carbon storage traits

At the end of the drought treatment, on 31 July 2025, five seedlings of each species, *Q. variabilis* and *Q. mongolica*, were randomly selected from each drought treatment as biological replicates. Mature, fully expanded leaves were collected from each seedling. After being transported to the laboratory, the leaf samples were heated at 105 °C for 30 min to terminate enzymatic activity and then oven-dried at 65 °C for 48 h to constant mass. The dried samples were ground into a fine powder and sieved. The contents of soluble sugar and starch were determined by spectrophotometric determination method (Hewitt, 1958). Briefly, 0.15 g of dried leaf powder was extracted with 7 mL of 80% ethanol at 80 °C for 30 min, followed by centrifugation at 4,000 rpm for 10 min. The extraction was repeated twice, and the supernatants were pooled. Soluble sugar content was determined using the anthrone–sulfuric acid colorimetric method by measuring the absorbance at 620 nm with a spectrophotometer (721G, Jingke Technology Instrument Co., Ltd., Shanghai, China). The residue remaining after soluble sugar extraction was used for starch determination. A 2-mL aliquot of deionized water was added to the residue, which was then heated in a boiling water bath for 15 min. After the mixture was cooled, 2 mL of 9.2 mol L^-1^ HClO_4_ was added, and the mixture was shaken for 10 min to hydrolyze the starch. Subsequently, 4 mL of deionized water was added, and the mixture was centrifuged at 4,000 rpm for 10 min. The supernatant was transferred to a 50-mL volumetric flask. The hydrolysis procedure was repeated using 4.6 mol L^-1^ HClO_4_. The resulting supernatant was pooled with the previously collected supernatant. The glucose of soluble sugars released from starch hydrolysis was determined spectrophotometrically. The starch content was estimated by multiplying the glucose concentration by a conversion factor of 0.9 (Bordoloi et al., 2012). Non-structural carbohydrate (NSC) content was calculated as the sum of soluble sugar and starch contents using **Equation 5**, and the soluble sugar-to-starch ratio (SS:ST) was calculated using **Equation 6**.

(5) NSC=SS+ST

(6) $$SS:ST=\frac{SS}{ST}$$

where NSC represents non-structural carbohydrate content, SS represents soluble sugar content, and ST represents starch content.

#### 2.2.5 Stress defense traits

Chlorophyll (Chl) content was determined using the acetone extraction method (Wellburn, 1994), and malondialdehyde (MDA) content was determined using the thiobarbituric acid method (Heath and Packer, 1968). Proline (Pro) content was measured using the ninhydrin colorimetric method (Bates et al., 1973). Peroxidase (POD) activity was determined using the guaiacol colorimetric method (Chance and Maehly, 1955), superoxide dismutase (SOD) activity using the nitroblue tetrazolium method (Giannopolitis and Ries, 1977), and catalase (CAT) activity using ultraviolet spectrophotometry (Aebi, 1984).

#### 2.2.6 Nutrient stoichiometric traits

Leaf total carbon (TC) content was determined using the potassium dichromate external-heating titration method (Bremner and Jenkinson, 1960). Leaf total nitrogen (TN) content was measured using a Kjeldahl nitrogen analyzer after digestion with sulfuric acid and hydrogen peroxide (Parkinson and Allen, 1975). Leaf total phosphorus (TP) content was determined using the molybdenum–antimony colorimetric method after digestion with sulfuric acid and perchloric acid (Cresser and Parsons, 1979).

### 2.3 Statistical analysis

Raw data were compiled and checked in Microsoft Excel 2019, whereas statistical analyses and data visualization were conducted in R version 4.1.2. The distributional properties and homogeneity of variance of each response variable were assessed before hypothesis testing. Variables were transformed when necessary, and non-parametric procedures were used when the assumptions of parametric tests could not be adequately satisfied. For each oak species, variation in individual functional traits among the four water treatments was examined using one-way analysis of variance (ANOVA), followed by Tukey’s honestly significant difference test for multiple comparisons. Differences between the two species were evaluated within each water treatment using independent-samples t-tests. Data are presented as means ± standard errors (SE). Treatment means sharing the same letter did not differ significantly, whereas different letters indicate significant differences at *P* < 0.05. Pairwise associations among leaf traits were quantified using Pearson’s correlation coefficients. Spearman’s rank correlation was used when the assumptions underlying Pearson’s correlation were not satisfied. Principal component analysis (PCA) was used to summarize multivariate patterns of leaf trait variation and identify the dominant dimensions of drought response and the major trait associations characterizing each species. To ensure comparability among variables measured in different units, all traits were z-standardized before PCA.

Species-specific coordination among functional trait groups was further investigated using PCA-based piecewise structural equation modeling (piecewise SEM). Separate models were fitted for *Q. variabilis* and *Q. mongolica*. In each model, experimentally imposed water availability (WATER) was included as an exogenous predictor and represented by the percentage of substrate field capacity assigned to each treatment. The remaining traits were classified into six biologically defined functional groups: hydraulic traits (HYD), stress-defense traits (DEF), photosynthetic and gas-exchange traits (GEX), carbon-storage traits (CAR), nutrient stoichiometry (NUT), and leaf morphological traits (LST). Within each species, dimensionality reduction was performed separately for each functional group, and the resulting PC1 scores were used as module-level composite variables in the path models. Overall model fit was evaluated using Fisher’s C statistic. Candidate model structures were ranked according to the Akaike information criterion (AIC), with lower values indicating greater relative support. The final path structure was selected by jointly considering prior ecological hypotheses, overall model fit, estimated path relationships, and the biological interpretability of the resulting model.

## 3 Results

### 3.1 Leaf functional traits of *Q. variabilis* and *Q. mongolica* seedlings under different drought treatments

Drought treatments significantly affected leaf biomass, leaf area, and specific leaf area in *Q. variabilis* seedlings (*P* < 0.05). Leaf biomass and leaf area in *Q. variabilis* reached their highest values under light drought (LD). In *Q. mongolica*, leaf biomass, leaf area, and specific leaf area declined significantly with increasing drought intensity (*P* < 0.05). Under the control and LD treatments, *Q. variabilis* seedlings had significantly greater leaf biomass and leaf area than *Q. mongolica* seedlings. By contrast, specific leaf area was significantly higher in *Q. mongolica* than in Q. variabilis under the control treatment, but decreased progressively in *Q. mongolica* as drought intensity increased (**Figure 2**).

**Figure 2:**
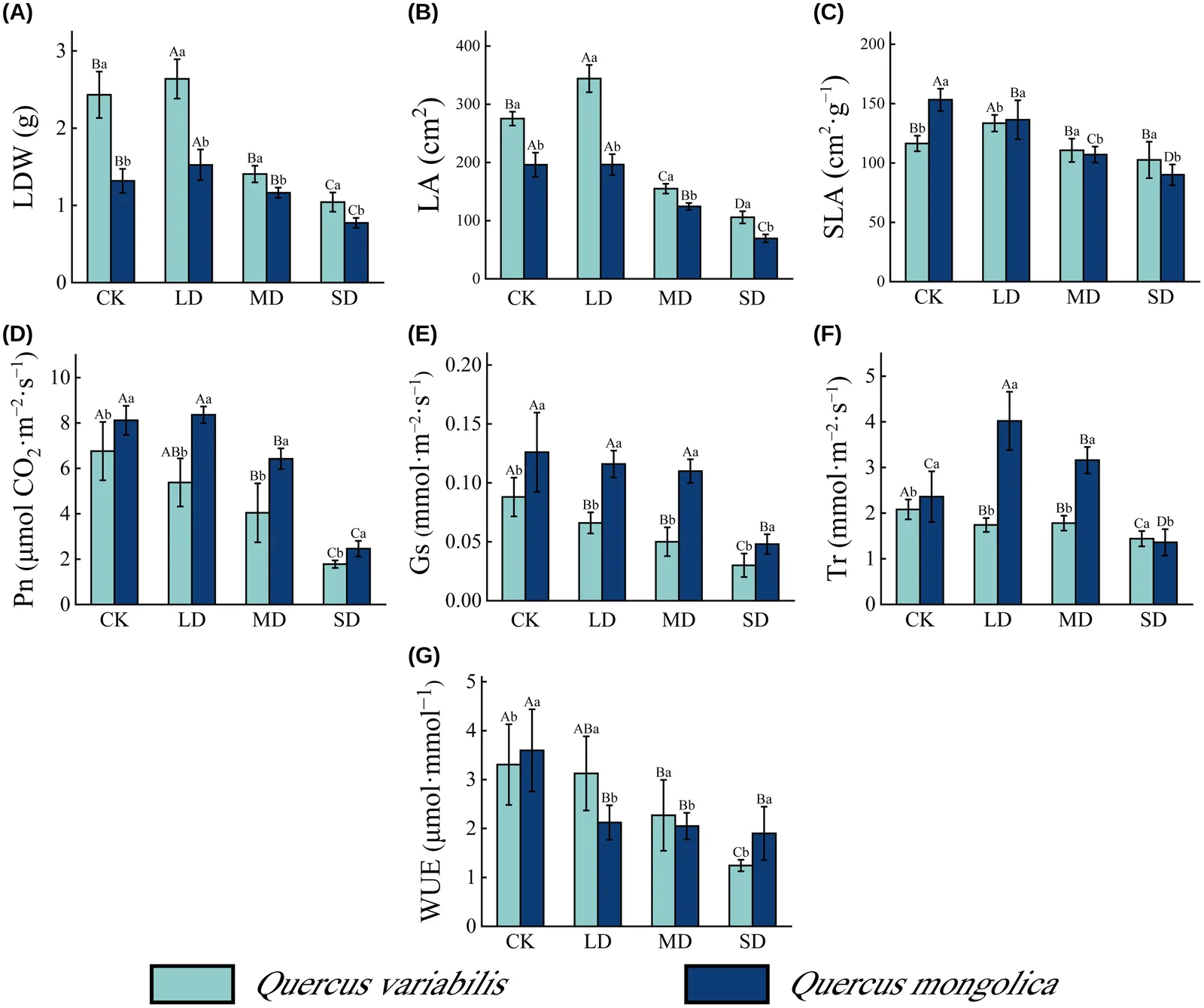
Leaf morphological traits and photosynthetic and gas-exchange parameters of *Q. variabilis* and *Q. mongolica* seedlings under drought stress. **(A)** Leaf dry weight (LDW); **(B)** leaf area (LA); **(C)** specific leaf area (SLA); **(D)** net photosynthetic rate (Pn); **(E)** stomatal conductance (Gs); **(F)** transpiration rate (Tr); and **(G)** water-use efficiency (WUE). CK, LD, MD, and SD denote the control, mild drought, moderate drought, and severe drought treatments, respectively. Bars represent means ± standard errors (SE; n = 5). Different uppercase letters indicate significant differences among drought treatments within the same species, whereas different lowercase letters indicate significant differences between the two species within the same drought treatment. Differences among drought treatments were assessed using one-way analysis of variance (ANOVA), and differences between species were assessed using independent-samples t-tests. Bars sharing the same letter are not significantly different, whereas bars with different letters differ significantly (P < 0.05).

In this study, drought stress significantly altered photosynthetic traits in both *Q. variabilis* and *Q. mongolica* seedlings. *In Q. variabilis*, net photosynthetic rate (Pn), stomatal conductance (Gs), transpiration rate (Tr), and water use efficiency (WUE) decreased significantly with increasing drought severity *(P* < 0.05). In *Q. mongolica*, Pn, Gs, and Tr also decreased significantly under increasing drought stress (*P* < 0.05), whereas WUE showed no significant differences among the LD, MD, and SD treatments (*P* > 0.05). Across most drought treatments, *Q. mongolica* exhibited higher Pn, Gs, and Tr than *Q. variabilis* (**Figure 2**).

Hydraulic measurements showed progressive deterioration in both oak species with increasing drought intensity. Predawn and midday leaf water potentials became significantly more negative, leaf relative water content decreased significantly, and stem embolism increased significantly in both *Q. variabilis* and *Q. mongolica* seedlings (*P* < 0.05). Under mild drought, both species maintained relatively stable hydraulic status, with only slight decreases in leaf water potential and relative water content and a limited increase in stem embolism. Under moderate drought, stem embolism increased markedly. Under severe drought, predawn and midday leaf water potentials became sharply more negative in both species, and stem embolism reached its highest levels. Species comparisons under severe drought showed a greater decrease in leaf water potential in *Q. mongolica* seedlings, but more extensive stem embolism in *Q. variabilis* seedlings. In contrast, differences in leaf relative water content between the two species remained small across drought treatments (**Figure 3**).

**Figure 3:**
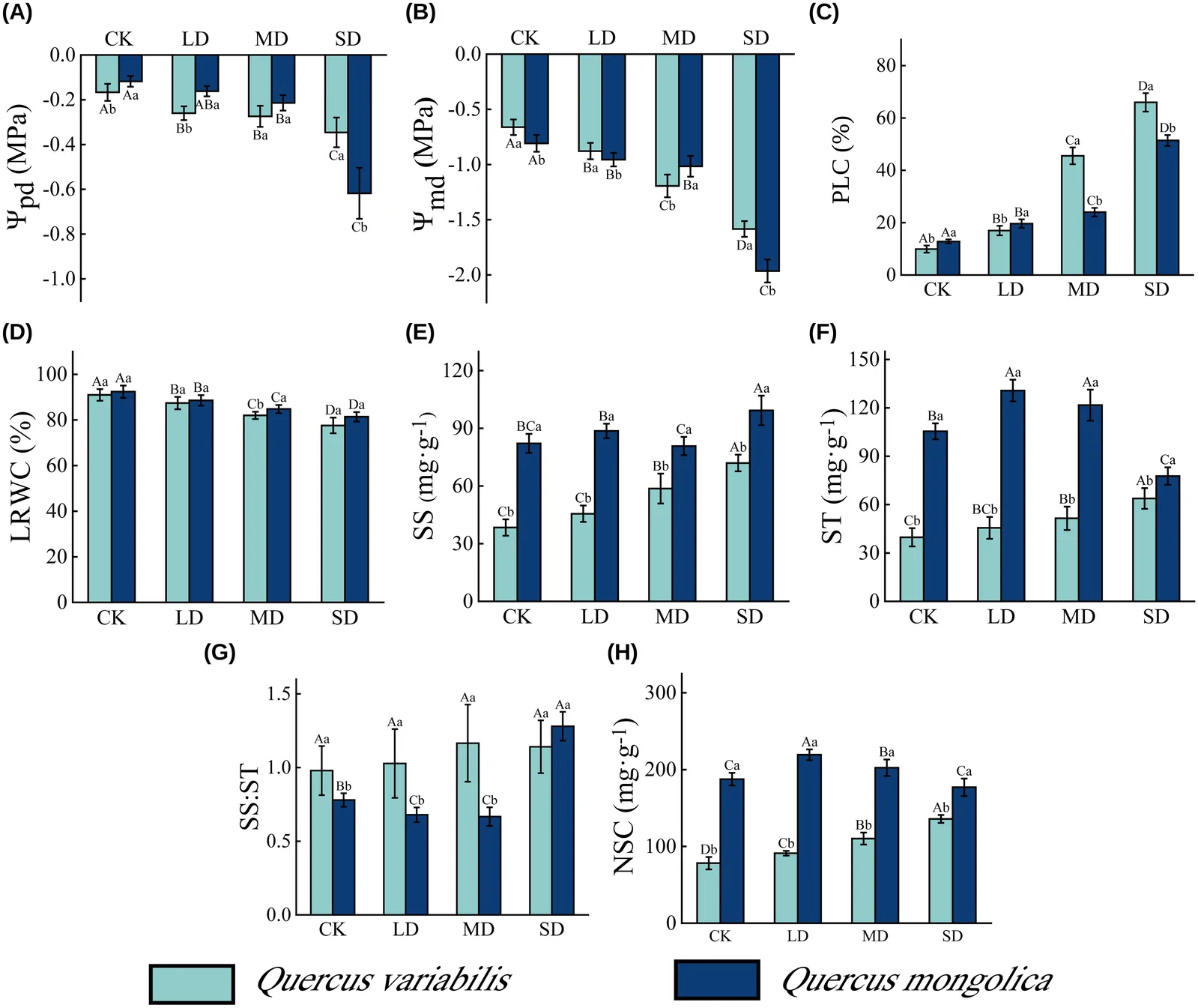
Hydraulic and carbon-storage traits of *Q. variabilis* and *Q. mongolica* seedlings under drought stress. **(A)** Predawn leaf water potential (Ψpd); **(B)** midday leaf water potential (Ψmd); **(C)** stem percentage loss of conductivity (PLC); **(D)** leaf relative water content (LRWC); **(E)** soluble sugar content (SS); **(F)** starch content (ST); **(G)** soluble sugar-to-starch ratio (SS: ST); and **(H)** non-structural carbohydrate content (NSC). CK, LD, MD, and SD denote the control, mild drought, moderate drought, and severe drought treatments, respectively. Bars represent means ± standard errors (SE; n = 5). Different uppercase letters indicate significant differences among drought treatments within the same species, whereas different lowercase letters indicate significant differences between the two species within the same drought treatment. Differences among drought treatments were assessed using one-way analysis of variance (ANOVA), and differences between species were assessed using independent-samples t-tests. Bars sharing the same letter are not significantly different, whereas bars with different letters differ significantly (*P* < 0.05).

Leaf carbon storage traits showed species-specific responses to drought. In *Q. variabilis*, leaf soluble sugar, starch, and non-structural carbohydrate (NSC) concentrations increased progressively with drought severity, reaching their highest levels under severe drought. The soluble sugar:starch ratio was generally greater than 1, indicating that soluble sugars dominated the leaf NSC pool. In *Q. mongolica*, soluble sugar concentration was highest under severe drought, whereas starch and total NSC concentrations peaked under mild drought and declined thereafter. The soluble sugar:starch ratio remained below 1 from the control to moderate drought treatments, but increased to around or above 1 under severe drought, indicating a shift in NSC composition. Across treatments, *Q. mongolica* generally maintained higher leaf NSC concentrations than *Q. variabilis*, although this difference narrowed under severe drought (**Figure 3**).

Drought treatment significantly affected leaf chlorophyll, malondialdehyde, and proline contents, as well as antioxidant enzyme activities, in seedlings of both *Quercus* species (*P* < 0.05). In *Q. variabilis*, leaf chlorophyll content declined significantly with increasing drought intensity (*P* < 0.05). In contrast, chlorophyll content in *Q. mongolica* increased under mild drought but decreased under moderate and severe drought. Leaf malondialdehyde and proline contents increased progressively with drought intensity in both species and were generally higher in *Q. variabilis* than in *Q. mongolica* across treatments. Catalase and superoxide dismutase activities were generally higher in *Q. variabilis* than in *Q. mongolica*. Catalase activity peaked under mild drought, whereas superoxide dismutase activity remained relatively high under both mild and moderate drought. By contrast, peroxidase activity was significantly higher in *Q. mongolica* than in *Q. variabilis* and reached its maximum under moderate drought. Overall, these results show that both *Quercus* species exhibited marked drought-induced changes in stress-defense traits, but differed in the magnitude of these responses and in the dominant antioxidant enzymes involved (**Figure 4**).

**Figure 4:**
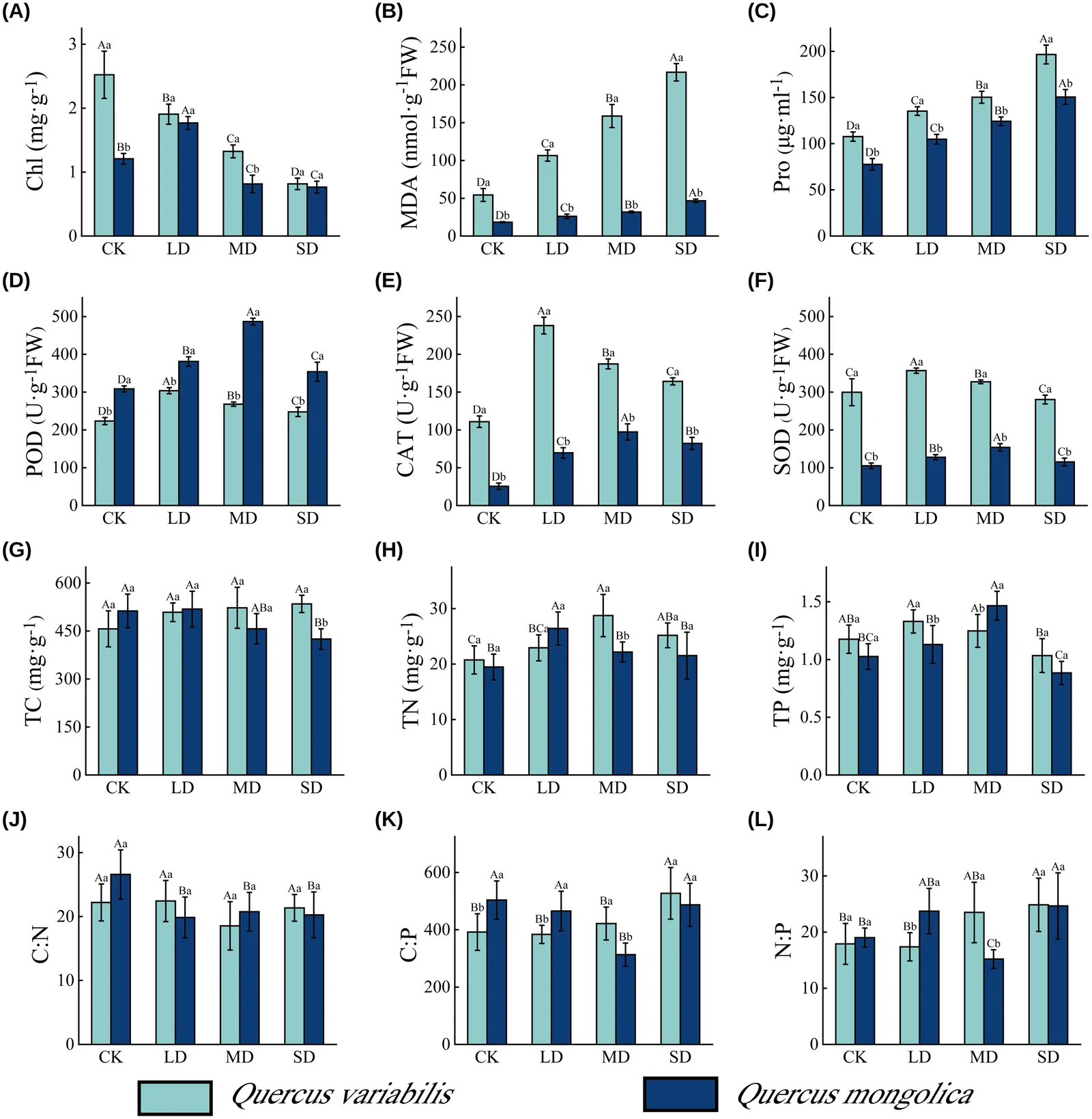
Stress-defense and nutrient stoichiometric traits of *Q. variabilis* and *Q. mongolica* seedlings under drought stress. **(A)** Chlorophyll content (Chl); **(B)** malondialdehyde content (MDA); **(C)** proline content (Pro); **(D)** peroxidase activity (POD); **(E)** catalase activity (CAT); **(F)** superoxide dismutase activity (SOD); **(G)** total carbon content (TC); **(H)** total nitrogen content (TN); **(I)** total phosphorus content (TP); **(J)** carbon-to-nitrogen ratio (C:N); **(K)** carbon-to-phosphorus ratio (C:P); and **(L)** nitrogen-to-phosphorus ratio (N:P).

In terms of nutrient stoichiometry, drought stress differentially affected leaf total carbon (TC), total nitrogen (TN), and total phosphorus (TP) concentrations, as well as their stoichiometric ratios, in *Q. variabilis* and *Q. mongolica* seedlings. Leaf TC concentration in *Q. variabilis* did not differ significantly among drought treatments, whereas that in *Q. mongolica* decreased with increasing drought severity and was significantly lower under SD than under CK and LD. Leaf TN concentration in both species initially increased and then decreased, reaching its maximum under MD in *Q. variabilis* and under LD in *Q. mongolica*. Leaf TP concentration decreased under SD in both species and was significantly higher in *Q. variabilis* than in *Q. mongolica* under LD (**Figure 4**).

For stoichiometric ratios, the leaf C:N ratio in *Q. variabilis* did not differ significantly among treatments, whereas that in *Q. mongolica* was generally lower under drought treatments than under CK. However, no significant interspecific differences in leaf C:N ratio were detected within the same drought treatment. The leaf N:P ratio in *Q. variabilis* generally increased with increasing drought severity, whereas that in *Q. mongolica* showed no consistent treatment-related pattern. Under LD, the leaf N:P ratio was significantly higher in Q. mongolica than in *Q. variabilis*, whereas the opposite pattern was observed under MD. The leaf C:P ratio in *Q. variabilis* reached its maximum under SD, whereas that in *Q. mongolica* reached its minimum under MD. Under CK and LD, the leaf C:P ratio was significantly higher in *Q. mongolica* than in *Q. variabilis*, whereas under MD it was significantly higher in *Q. variabilis* than in *Q. mongolica*.

### 3.2 Correlations among leaf functional traits in *Q. variabilis* and *Q. mongolica* seedlings

Pearson correlation analysis showed that multiple leaf functional traits were significantly correlated in both oak species, although the correlation structure differed between species. Using a significance threshold of *P* < 0.05, 224 significant trait-pair correlations were detected in *Q. variabilis* seedlings, whereas 211 were detected in *Q. mongolica* seedlings, indicating slightly stronger overall leaf trait covariation in *Q. variabilis* than in *Q. mongolica* (**Figure 5**).

**Figure 5:**
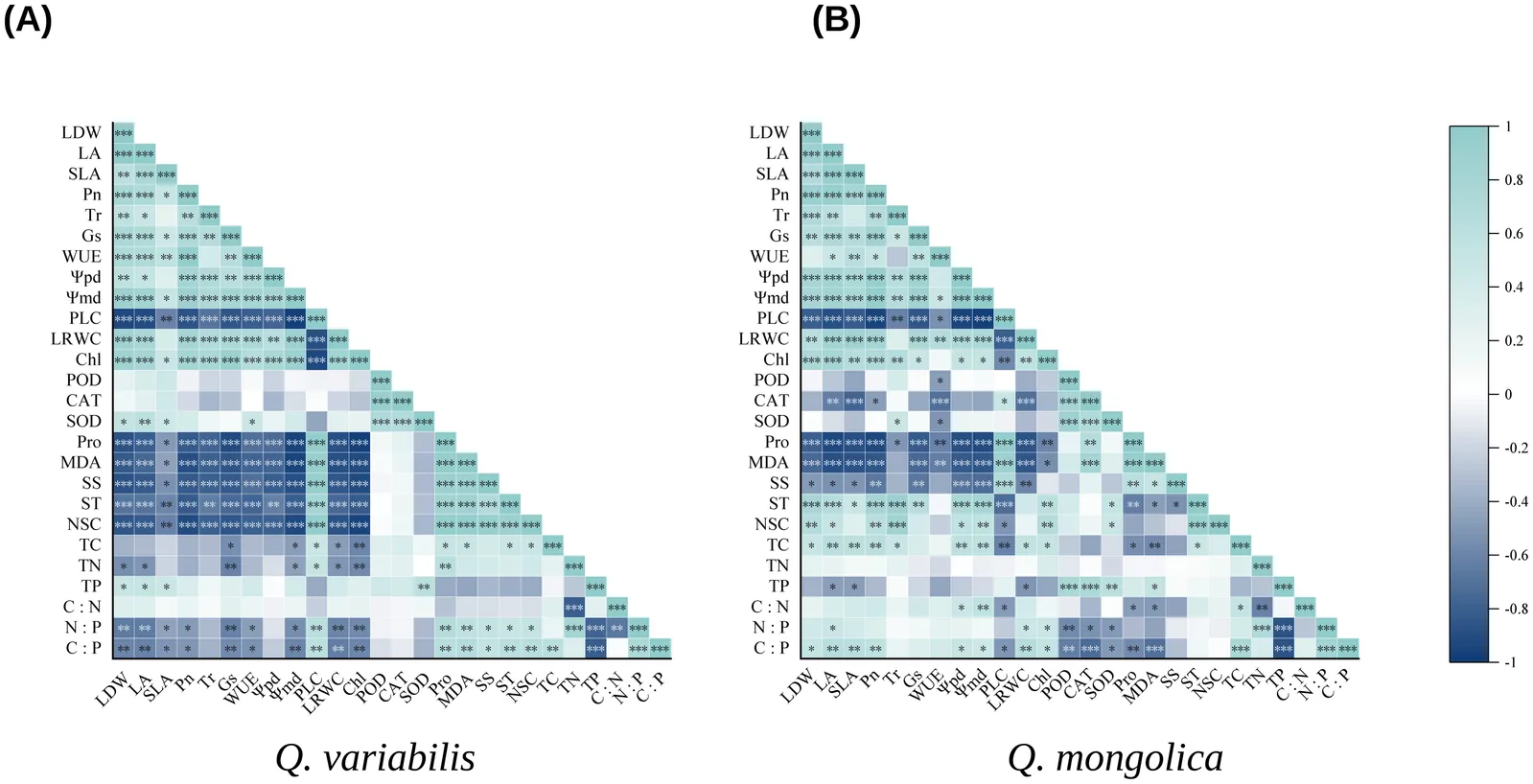
Pearson correlations among leaf traits of *Q. variabilis* and *Q. mongolica* seedlings under different drought-stress treatments. **(A)** *Q. variabilis*; **(B)** *Q. mongolica*. The color scale represents Pearson’s correlation coefficient (r), ranging from −1 to 1, with positive and negative values indicating positive and negative correlations, respectively. Asterisks indicate statistically significant correlations: P < 0.05, P < 0.01, and P < 0.001. LDW, leaf dry biomass; LA, leaf area; SLA, specific leaf area; Pn, net photosynthetic rate; Tr, transpiration rate; Gs, stomatal conductance; WUE, water-use efficiency; Ψpd, predawn leaf water potential; Ψmd, midday leaf water potential; PLC, percentage loss of hydraulic conductivity; LRWC, leaf relative water content; Chl, chlorophyll content; POD, peroxidase; CAT, catalase; SOD, superoxide dismutase; Pro, proline; MDA, malondialdehyde; SS, soluble sugars; ST, starch; NSC, non-structural carbohydrates; TC, total leaf carbon; TN, total leaf nitrogen; TP, total leaf phosphorus.

In *Q. variabilis* seedlings, leaf non-structural carbohydrate (NSC) content was strongly and positively correlated with malondialdehyde (MDA), proline, soluble sugar, starch, and percentage loss of stem hydraulic conductivity (PLC) (*P* < 0.001). By contrast, leaf NSC content was strongly and negatively correlated with leaf dry weight, leaf area, net photosynthetic rate, stomatal conductance, transpiration rate, water-use efficiency, predawn leaf water potential, midday leaf water potential, and chlorophyll content (*P* < 0.001). These correlations indicate that variation in leaf NSC content in *Q. variabilis* was closely associated with stress-related biochemical responses and hydraulic impairment, but inversely associated with leaf growth and photosynthetic gas exchange.

In *Q. mongolica* seedlings, leaf NSC content was strongly and positively correlated with stomatal conductance and starch content (*P* < 0.001), positively correlated with leaf dry weight, net photosynthetic rate, predawn leaf water potential, and midday leaf water potential (*P* < 0.01), and negatively correlated with PLC (*P* < 0.05). Leaf NSC content was also significantly correlated with leaf area and superoxide dismutase activity. Compared with *Q. variabilis*, *Q. mongolica* showed stronger positive correlations between NSC content and photosynthetic gas exchange, leaf water status, and carbon storage traits. Several other trait pairs showed weak or nonsignificant correlations, indicating limited linear covariation among these traits.

### 3.3 Principal component analysis of leaf functional traits in *Q. variabilis* and *Q. mongolica* seedlings

Principal component analysis (PCA) showed that the first two principal components explained 73.4% of the total variation in leaf functional traits of *Q. variabilis* seedlings, with PC1 and PC2 accounting for 58.8% and 14.6%, respectively. For *Q. mongolica* seedlings, PC1 and PC2 together explained 73.0% of the total variation, accounting for 49.6% and 23.4%, respectively. Thus, the first two principal components captured most of the variation in leaf functional traits in both oak species.

In *Q. variabilis* seedlings, PC1, which explained 58.8% of the total variation, had high loadings for photosynthetic rate, chlorophyll content, midday leaf water potential, percentage loss of stem conductivity, malondialdehyde, and nonstructural carbohydrates (NSC). This axis contrasted traits related to growth, photosynthetic capacity, and plant water status with those associated with drought-induced stress responses and carbon storage. PC2, which explained 14.6% of the total variation, was primarily associated with catalase, peroxidase, superoxide dismutase, total phosphorus, and the leaf N ratio, indicating variation in antioxidant defense and stoichiometric traits (**Figure 6**). In *Q. mongolica* seedlings, PC1, which explained 49.6% of the total variation, had high loadings for leaf area, photosynthetic rate, midday leaf water potential, malondialdehyde, proline, and percentage loss of stem conductivity. PC2, which explained 23.4% of the total variation, loaded strongly on stomatal conductance, catalase, peroxidase, superoxide dismutase, starch, NSC, total phosphorus, and the leaf N ratio, indicating covariation among stomatal regulation, carbon storage, antioxidant defense, and stoichiometric traits (**Figure 6**).

**Figure 6:**
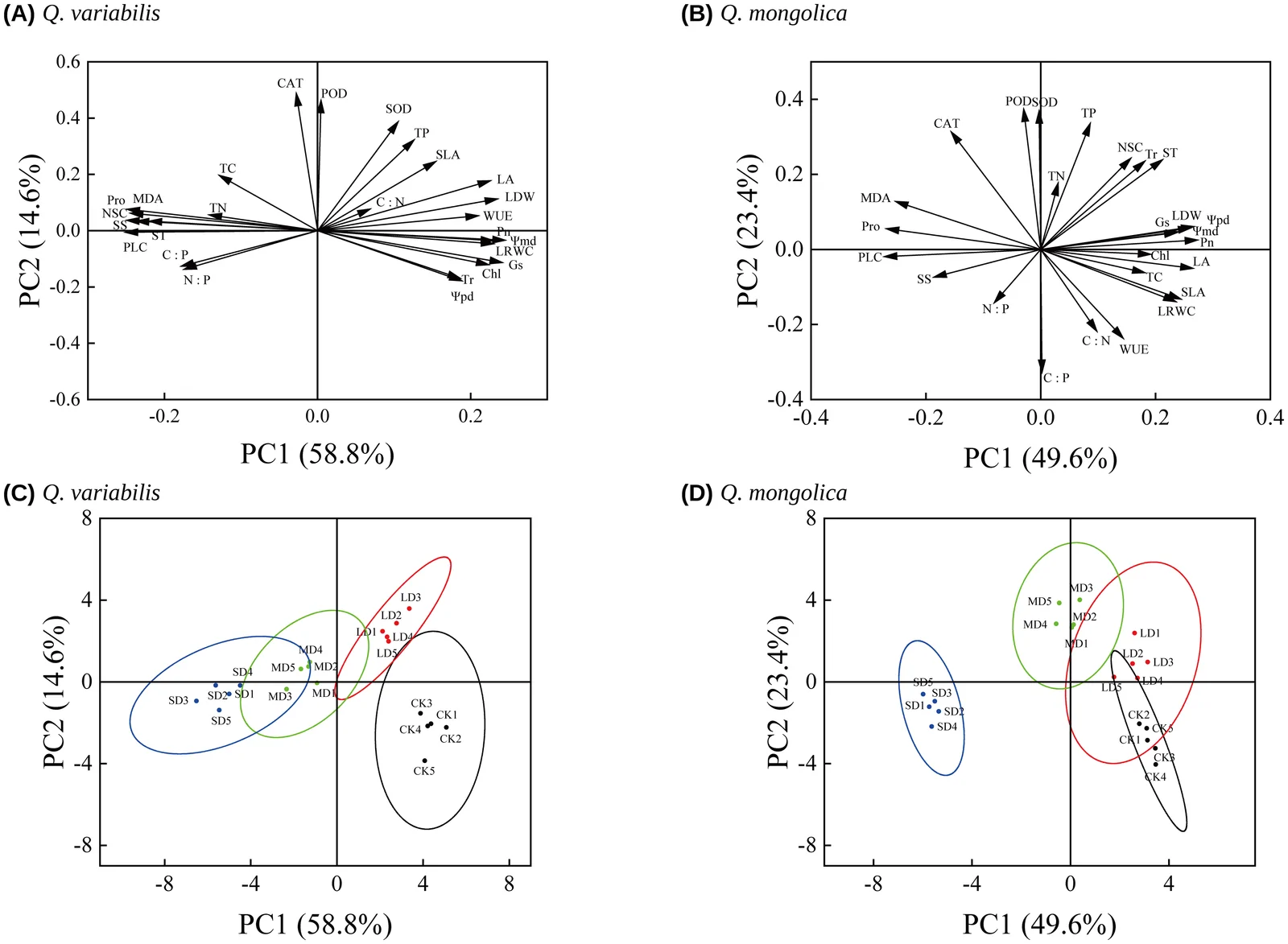
Principal component analysis (PCA) showing interspecific differences in leaf-trait relationships between *Q. variabilis* and *Q. mongolica* seedlings. **(A, B)** Trait-loading plots for *Q. variabilis* and *Q. mongolica*, respectively. The direction and length of each arrow indicate the direction and strength, respectively, of the association between the corresponding trait and the principal component axes. **(C, D)** PCA score plots showing the distribution of biological replicates of *Q. variabilis* and *Q. mongolica*, respectively, under the well-watered control and three drought treatments. LDW, leaf dry weight; LA, leaf area; SLA, specific leaf area; Pn, net photosynthetic rate; Tr, transpiration rate; Gs, stomatal conductance; WUE, water-use efficiency; Ψpd, predawn leaf water potential; Ψmd, midday leaf water potential; PLC, percentage loss of conductivity; LRWC, leaf relative water content; Chl, chlorophyll content; POD, peroxidase activity; CAT, catalase activity; SOD, superoxide dismutase activity; Pro, proline content; MDA, malondialdehyde content; SS, soluble sugar content; ST, starch content; NSC, non-structural carbohydrate content; TC, total leaf carbon concentration; TN, total leaf nitrogen concentration; TP, total leaf phosphorus concentration. CK, well-watered control; LD, mild drought; MD, moderate drought; SD, severe drought.

Overall, PCA ordination separated the drought treatments in both oak species, although the loading patterns of key traits differed between species. In *Q. variabilis* seedlings, NSC, together with soluble sugar, starch, malondialdehyde, proline, and percentage loss of stem conductivity, was located mainly in the negative region of PC1 and was oriented opposite to traits related to growth, photosynthesis, and plant water status. In *Q. mongolica* seedlings, NSC and starch showed strong loadings on PC2 and were oriented in a similar direction to stomatal conductance and antioxidant enzyme traits. These results indicate that the coordination between leaf NSC and other functional traits differed between the two oak species, reflecting species-specific patterns of trait covariation under drought stress.

### 3.4 Structural equation modelling of leaf functional traits in *Q. variabilis* and *Q. mongolica* seedlings

In the *Q. variabilis* seedling model, hydraulic traits had positive paths to stress-defense traits (β = 0.67, *p* < 0.05) and photosynthetic and gas-exchange traits (β = 0.65, *p* < 0.05). Photosynthetic and gas-exchange traits showed a significant negative path to carbon-storage traits (β = −0.58, *p* < 0.05), which in turn showed a significant negative path to leaf morphological traits (β = −0.87, *p* < 0.05). By contrast, the direct path from nutrient stoichiometric traits to leaf morphological traits was not significant. The model explained 95% of the variance in leaf morphological traits, suggesting that hydraulic, stress-defense, photosynthetic and gas-exchange, and carbon-storage traits jointly captured most of the variation in leaf morphology in *Q. variabilis* seedlings (**Figure 7**).

**Figure 7:**
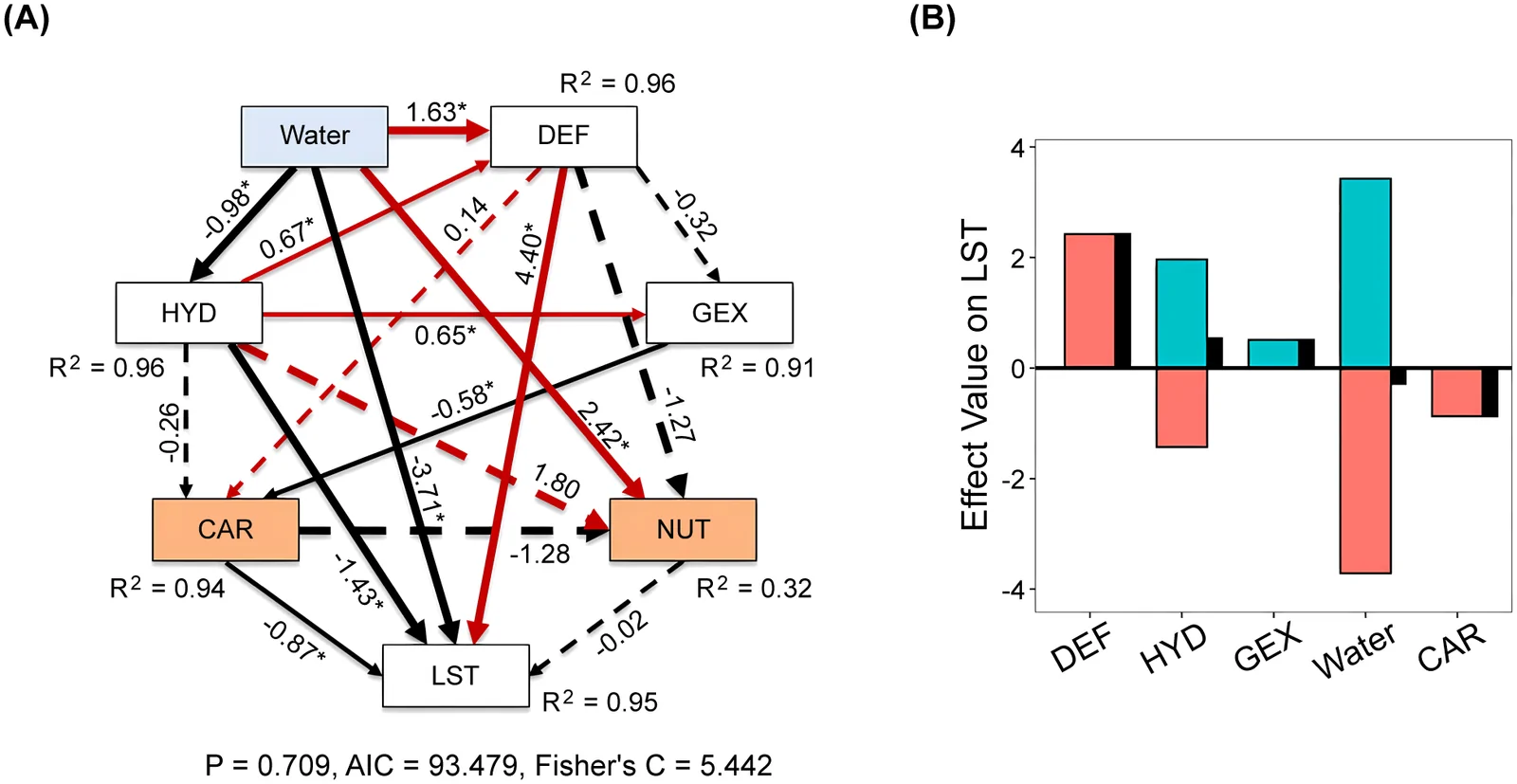
Structural equation modeling of leaf traits in *Q. variabilis* seedlings. **(A)** Structural equation model (SEM) for leaf traits. **(B)** Direct, indirect, and total effects of the predictor variables on LST. Water denotes the exogenous variable representing the water-availability gradient. HYD, DEF, GEX, CAR, NUT, and LST denote the first principal component (PC1) scores derived from the corresponding trait groups: hydraulic traits, stress-defense traits, photosynthetic and gas-exchange traits, carbon-storage traits, nutrient-stoichiometric traits, and leaf structural traits, respectively. Values adjacent to the arrows are standardized path coefficients. Solid arrows indicate significant effects (P < 0.05), whereas dashed arrows indicate nonsignificant effects (P ≥ 0.05). Red and black arrows indicate positive and negative effects, respectively.

In the *Q. mongolica* seedling model, the water gradient showed a significant negative path to hydraulic traits (β = −0.92, p < 0.001). Hydraulic traits had positive paths to stress-defense traits (β = 1.07, p < 0.001) and photosynthetic and gas-exchange traits (β = 0.99, p < 0.001). In contrast to the *Q. variabilis* model, stress-defense traits showed a significant positive path to carbon-storage traits in *Q. mongolica* seedlings (β = 0.57, p < 0.01), whereas the direct paths from hydraulic traits and photosynthetic and gas-exchange traits to carbon-storage traits were not significant. Carbon-storage traits also showed a significant positive direct path to leaf morphological traits (β = 0.59, p < 0.001), whereas the direct paths from hydraulic, stress-defense, and nutrient stoichiometric traits to leaf morphological traits were not significant. The model explained 94% of the variance in leaf morphological traits, suggesting that hydraulic, stress-defense, and carbon-storage traits jointly captured most of the variation in leaf morphology in *Q. mongolica* seedlings. Carbon-storage traits were the only trait group with a significant direct path to leaf morphological traits, indicating that this component was the closest direct predictor of leaf morphology in the *Q. mongolica* model (**Figure 8**).

**Figure 8:**
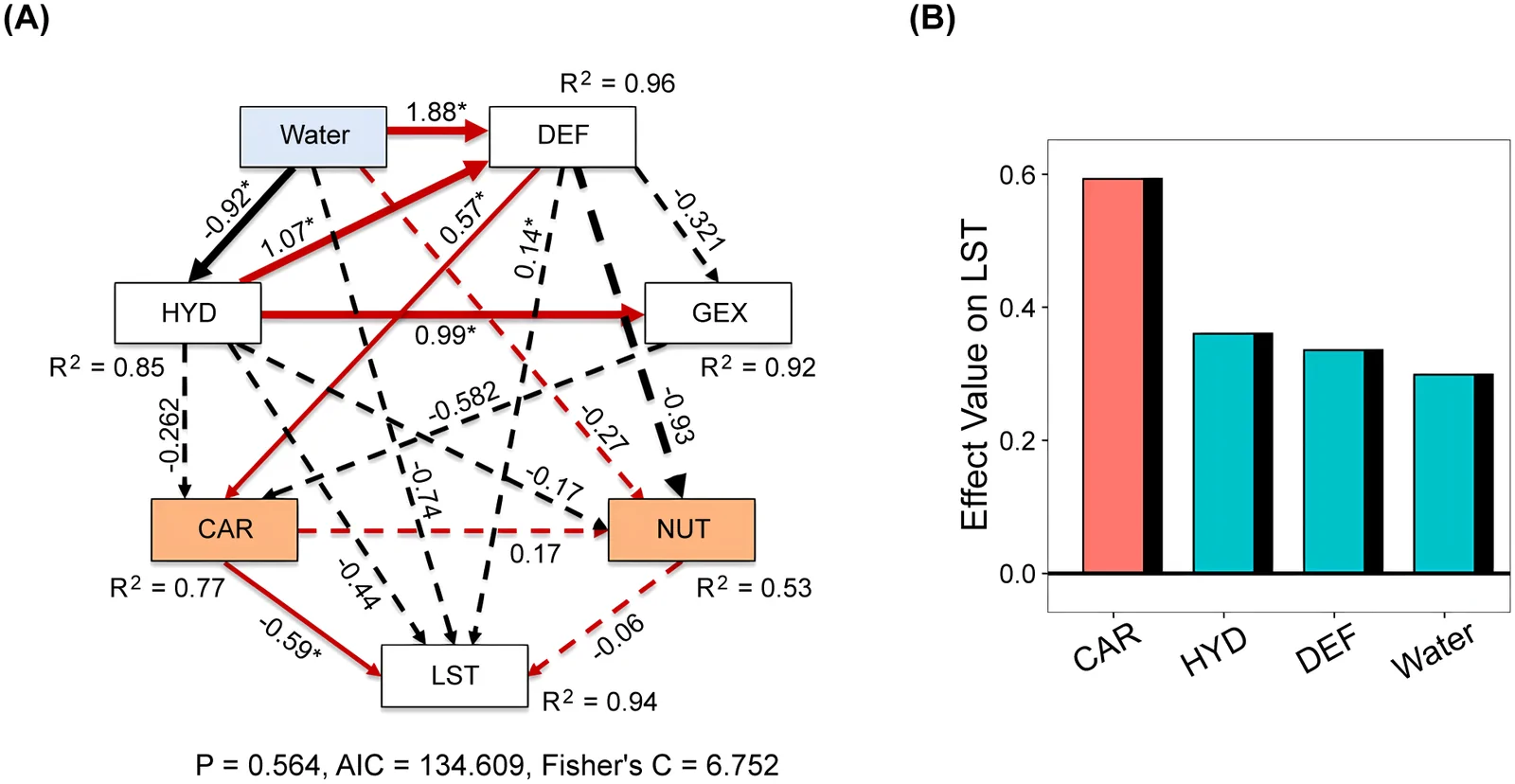
Structural equation model for leaf traits of *Q. mongolica* seedlings. **(A)** Structural equation model (SEM) for the leaf traits of *Q. mongolica* seedlings. **(B)** Direct, indirect, and total effects of the predictor variables on LST. Water denotes the exogenous variable representing the water-availability gradient. HYD, DEF, GEX, CAR, NUT, and LST denote the first principal component (PC1) scores derived from the corresponding trait groups: hydraulic traits, stress-defense traits, photosynthetic and gas-exchange traits, carbon-storage traits, nutrient-stoichiometric traits, and leaf structural traits, respectively. Values adjacent to the arrows are standardized path coefficients. Solid arrows indicate significant effects (*P* < 0.05), whereas dashed arrows indicate nonsignificant effects (*P* ≥ 0.05). Red and black arrows indicate positive and negative effects, respectively.

## 4 Discussion

Our findings reveal contrasting leaf-level drought-adaptation strategies between *Q. variabilis* and *Q. mongolica* seedlings. Previous studies have attributed interspecific variation in drought tolerance among oak species partly to differences in their ability to mitigate oxidative damage (Sobrino-Plata et al., 2024). Xiong et al. further showed that both antioxidant enzyme activity and its regulation differ among oak species (Xiong et al., 2022). Consistent with these findings, our results suggest that the divergent drought responses of the two species are primarily driven by species-specific changes in non-structural carbohydrate and their differential coordination with growth regulation and antioxidant defense.

In the leaf PCA of *Q. variabilis* seedlings, non-structural carbohydrates (NSC) showed a strong loading on the first principal component (PC1), which accounted for the largest proportion of the total variance. NSC was negatively associated with the morphological and physiological traits related to leaf growth that primarily defined this axis. This pattern suggests that variation in leaf NSC may be closely linked to carbon use for growth in *Q. variabilis* seedlings. Under mild drought or relatively favorable water conditions, recently assimilated carbon may be preferentially allocated to sustaining growth (Cho et al., 2022; Oswald and Aubrey, 2024). This preferential use of NSC for growth is consistent with findings for Artemisia ordosica in the Mu Us Sandy Land, where NSC also primarily supported growth under drought conditions (He et al., 2023). As drought intensifies, stomatal closure restricts CO_2_ diffusion into leaves and reduces photosynthesis. When growth is inhibited earlier or more strongly than photosynthesis, growth and photosynthesis may become decoupled, allowing part of the assimilated carbon to accumulate in leaves as NSC rather than being rapidly incorporated into structural biomass (Granda and Camarero, 2017; Urrutia-Jalabert et al., 2020; Blumstein et al., 2022). Thus, increased NSC under severe drought does not necessarily indicate carbon sufficiency; instead, it may reflect passive accumulation caused by growth limitation. This interpretation is consistent with studies showing that decoupling between growth and photosynthesis can promote NSC accumulation in plant tissues (O’Brien et al., 2015; Stefaniak et al., 2024). Therefore, although *Q. variabilis* seedling leaves accumulated NSC under severe drought, these reserves were not synchronously associated with structural growth or antioxidant defense when photosynthesis was constrained and growth was suppressed (Xiang et al., 2021; Wang et al., 2023; Oswald and Aubrey, 2024). Overall, leaf NSC in *Q. variabilis* seedlings appears to be mainly involved in growth maintenance and in carbon accumulation following growth limitation, rather than being tightly coupled with antioxidant responses. This interpretation also explains why NSC and antioxidant enzymes were separated on different principal components in the PCA. The SEM results further support this inference, indicating that hydraulic traits, stress-defense traits, and carbon-storage traits may influence leaf morphological traits through relatively independent pathways. In particular, the direct pathway from carbon-storage traits to leaf morphological traits suggests that NSC is closely associated with leaf growth regulation in *Q. variabilis* seedlings (Piper and Paula, 2020; Wang H et al., 2024).

In contrast to *Q. variabilis* seedlings, PCA based on leaf traits of *Q. mongolica* seedlings showed that non-structural carbohydrate (NSC) concentrations, stomatal conductance, and antioxidant enzyme activities loaded strongly on the second principal component (PC2) and changed in a broadly coordinated manner. This pattern suggests a drought-adaptation strategy in *Q. mongolica* leaves in which stomatal regulation, carbon storage, and antioxidant defense are closely coupled. Under drought stress, *Q. mongolica* leaves appear to maintain partial stomatal opening, which may help sustain CO_2_ diffusion and support continued photosynthetic carbon assimilation (Zhu et al., 2021; Zhang H et al., 2021). This response is consistent with previous findings that some *Quercus* species show less stringent stomatal closure under drought than conifers (Walthert et al., 2024). as drought stress intensifies, a fraction of newly assimilated carbon may not be immediately allocated to structural growth or respiration, but may instead accumulate in leaves as NSCs, particularly starch. This pattern is consistent with previous studies showing that plants can maintain carbon reserves under drought by reducing growth investment (Piper et al., 2017; Dickman et al., 2018). Meanwhile, the maintenance of photosynthetic activity during drought may increase the risk of reactive oxygen species (ROS) production through electron leakage from the photosynthetic electron transport chain. Enhanced antioxidant enzyme activity is therefore required to scavenge ROS and reduce the risk of membrane lipid peroxidation (Contran et al., 2013). mechanistically, NSCs may support antioxidant responses through multiple metabolic and signaling pathways (Afzal et al., 2021). First, soluble sugars can enter glycolysis and the tricarboxylic acid cycle, thereby providing carbon skeletons for amino acid biosynthesis and ATP for the synthesis and turnover of antioxidant enzymes (Dumont and Rivoal, 2019; Zheng et al., 2025). Second, glucose-6-phosphate can enter the pentose phosphate pathway to generate NADPH, which serves as an electron donor for the ascorbate–glutathione cycle and helps maintain the reducing capacity of the antioxidant system (Wang et al., 2016; Foyer and Kunert, 2024). In addition, soluble sugars such as sucrose and glucose can act as signaling molecules that activate stress-responsive genes, including those encoding antioxidant enzymes, thereby modulating defense capacity at the transcriptional level (Wang et al., 2020; Jeandet et al., 2022). Taken together, NSCs in *Q. mongolica* seedling leaves may be closely linked to stomatal behavior and antioxidant defense, suggesting that their primary role under drought stress is more likely to support defense-related processes than to directly promote growth. The structural equation model is consistent with this inference, indicating that hydraulic and stress-defense traits are associated with carbon-storage traits, which in turn are linked to leaf growth. In this pathway, carbon storage occupies a central position, suggesting that drought-induced changes in hydraulic status and defense capacity may regulate leaf growth partly through their effects on carbon storage. differential accumulation of soluble sugars and starch within the NSC pool may further support stress-defense processes at multiple levels, including stress signaling, redox regulation, and reducing-power supply (Thalmann and Santelia, 2017; AbdElgawad et al., 2020; Ma et al., 2024). Overall, *Q. mongolica* seedling leaves exhibit a defense-prioritized resource-allocation pattern under drought stress, in which carbon reserves and associated defense processes are maintained rather than carbon being preferentially allocated to immediate growth.

Within the ecotone, the divergent drought-adaptation strategies of *Q. variabilis* and *Q. mongolica* seedlings may reflect niche differentiation and species coexistence mediated by trait trade-offs (Kunstler et al., 2016; Pérez-Ramos et al., 2019). From an evolutionary ecological perspective, this divergence is likely linked to the long-term hydrothermal regimes under which the two species have evolved. *Q. variabilis* is distributed mainly across warm-temperate to subtropical regions, where habitats are typically characterized by longer growing seasons and stronger competition for resources (Liu et al., 2018; Wang et al., 2024a). When water availability is relatively high, allocating more carbon to rapid growth can enhance access to space and light, potentially favoring trait combinations associated with growth maintenance and rapid carbon use (Wilcox et al., 2020; Joswig et al., 2021). By contrast, *Q. mongolica* is more widely distributed in temperate and cold-temperate regions and has long been exposed to environmental constraints such as low temperature and seasonal drought (Zhang X et al., 2021). In such environments, maintaining greater carbon reserves and strengthening survival-related processes, including antioxidant defense, may help this species preserve cellular function under stress rather than maximize rapid growth. From a coexistence perspective, the growth-prioritized strategy of *Q. variabilis* may support rapid biomass accumulation, light capture, and competitive advantage during wetter periods or in microhabitats with higher water availability (DeMalach et al., 2016). Under intensified drought and severe water deficit, however, the defense-prioritized strategy of *Q. mongolica* may reduce the risk of drought-induced injury and enhance persistence (Tao et al., 2024). These contrasting responses to environmental fluctuation provide a potential physiological and ecological mechanism for the coexistence of growth- and defense-prioritized strategies within the ecotone. They may further allow life-history strategies adapted to habitats differing in water availability to coexist within the regional species pool, thereby helping maintain functional response diversity at the community level (Sasaki et al., 2019).With drought events becoming increasingly frequent and severe, the strong coordination among carbon storage, stomatal regulation, and antioxidant defense observed in *Q. mongolica* seedlings in this study may help maintain carbon availability and physiological stability under water-limited conditions, consistent with previous studies showing drought-induced adjustments in NSC storage, stomatal regulation, and antioxidant responses in *Q. mongolica* seedlings (Wang et al., 2023; Yuan et al., 2025). However, whether these functional trait differences translate into higher whole-plant survival or regeneration success for *Q. mongolica* than for *Q. variabilis* under natural drought conditions remains uncertain and requires further investigation. Long-term seedling survival and regeneration depend not only on physiological drought tolerance but also on carbon balance, light availability, and other habitat conditions (Huo et al., 2024; Wu et al., 2024).

## 5 Conclusion

By integrating multidimensional functional traits related to leaf morphology, photosynthesis, hydraulics, carbon storage, antioxidant defense, and nutrient stoichiometry, this study revealed contrasting patterns of functional trait coordination and drought-adaptation strategies in *Q. variabilis* and *Q. mongolica* seedlings under drought stress. The main contribution of this study is to demonstrate that differences in drought responses between the two co-occurring oak species are primarily reflected in contrasting coordination patterns between non-structural carbohydrates and growth, stomatal regulation, and antioxidant defense. *Q. variabilis* seedlings exhibited a resource-allocation strategy relatively biased toward maintaining growth, whereas *Q. mongolica* seedlings showed a stronger tendency to prioritize stress defense, maintenance, and physiological stability. This divergence in functional trait coordination provides a new integrative trait-based perspective for understanding drought adaptation and niche differentiation in co-occurring oak seedlings within ecotonal regions and offers an ecophysiological basis for understanding potential changes in forest regeneration dynamics under increasingly severe drought conditions.

## Data Availability

The original contributions presented in the study are included in the article/**Supplementary Material**. Further inquiries can be directed to the corresponding authors.
